# Variable weights theory and its application to multi-attribute group decision making with intuitionistic fuzzy numbers on determining decision maker’s weights

**DOI:** 10.1371/journal.pone.0212636

**Published:** 2019-03-06

**Authors:** Sen Liu, Wei Yu, Ling Liu, Yanan Hu

**Affiliations:** 1 School of Logistics, Yunnan University of Finance and Economics, Kunming, China; 2 Business analytics department, Rajax network technology limited-liability company, Guangzhou, China; Northeast Electric Power University, CHINA

## Abstract

The determination of the weights of decision makers (DMs) is an important problem in multi-attribute group decision making. Many approaches have been presented to determine DMs’ weights. However, the computed weight vectors of DMs are usually assumed to be constant in existing studies, and this may cause irrationalities in the decision results. Therefore, this article proposes a novel method to determine DMs’ weights based on variable weights theory in which the evaluation information is described as intuitionistic fuzzy sets (IFSs). First, DMs provide their assessment with IFSs, and the intuitionistic fuzzy weighted averaging (IFWA) operator is applied to obtain weighted decision matrix based on the prior given DMs’ and attributes’ weights. Second, the DMs’ weights are obtained based on variable weights theory, and an alternative decision can be computed. Finally, the converted value of the achieved IFS of each alternative is calculated, and the best appropriate alternative is acquired. Two illustrative examples and the comparisons with exsiting approaches are also used to reflect the effectiveness of the proposed approach.

## 1 Introduction

Multi-attribute group decision making (MAGDM) is usually used to choose the best alternative from a group of ones according to the multi-attributes (also called criteria) [1]. The purpose is to help the decision maker (DM) in using a more efficient, rational and explicit decision tool to fully analyse all the important subjective and objective attributes of the problem [2–4]. To make the decision results more accurate, researchers began to use fuzzy set theory to study MAGDM problems, and which are widely used in many areas, such as supplier selection [5, 6], hub location [7] and heat and power economic emission dispatch [8], etc. As an important component of MAGDM, fuzzy multi-attribute group decision making (FMAGDM) is a difficult and hot research area [9].

As an extension of fuzzy sets [10], the intuitionistic fuzzy sets approach (IFS)[11] is a powerful instrument to deal with imprecise and imperfect data in MAGDM problems. In recent years, many scholars have researched IFSs and used them in many areas, such as decision making [12–14], image fusion [15], and other management problems [16–18].

**Fig 1:**
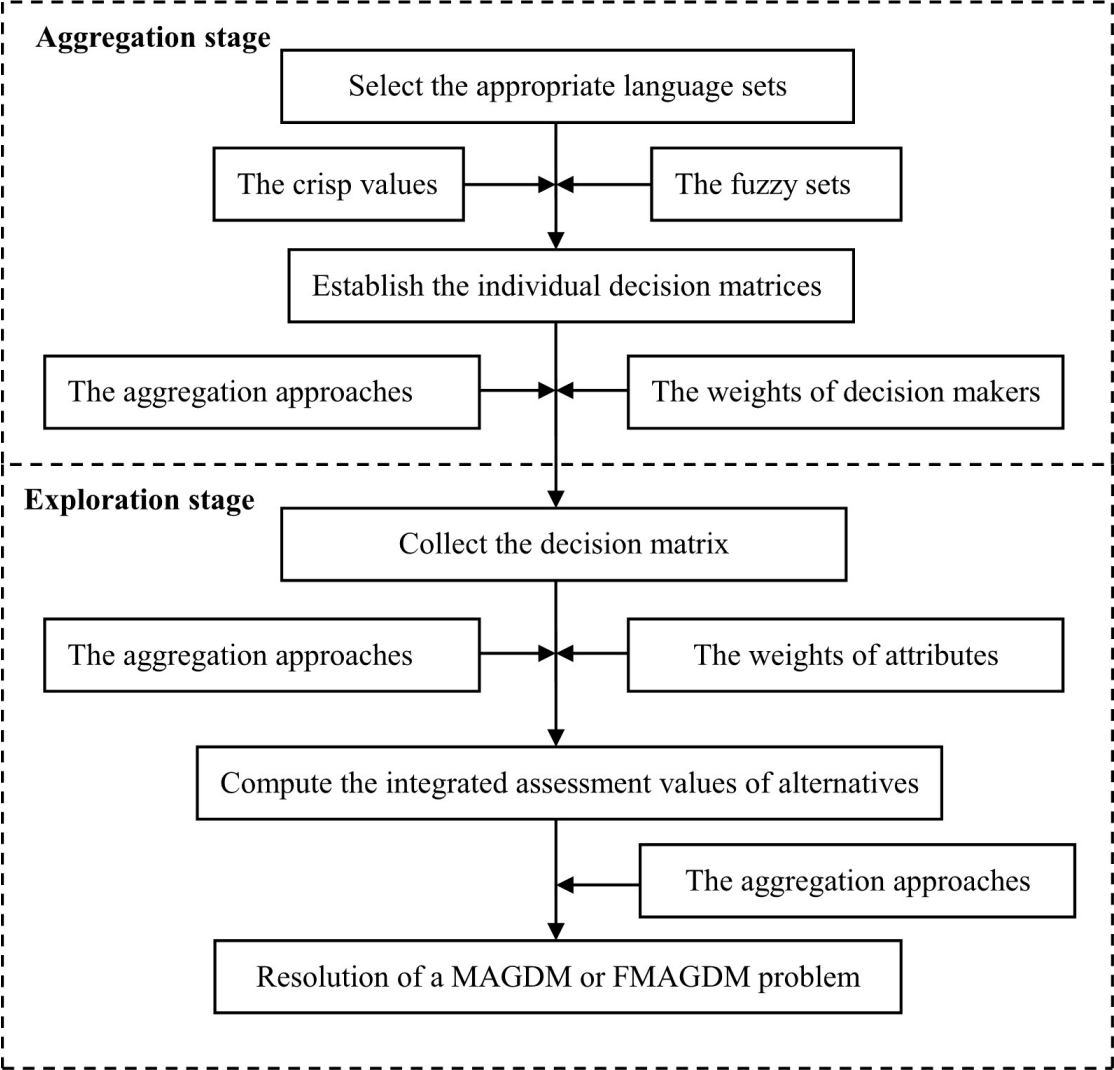
The process of resolving a MAGDM/ FMAGDM problem.

Usually, most the resolution of MAGDM and FMAGDM problem consists of two stages: aggregation and exploration [14, 19]. In the aggregation stage, top management select appropriate language sets based on crisp values or a variety of fuzzy sets, which leads to a MAGDM or FMAGDM problem. Then, the decision matrices are aggregated into a collective one with the weights of DMs. In the exploration stage, the collective decision matrix is converted into the integrated assessment values of alternatives with the weights of attributes and the various aggregation methods. Fig 1 illustrates the general process of resolving a MAGDM/ FMAGDM problem [14].

It is easy to see that MAGDM problems with IFSs usually have three common characteristics, including multiple DMs, alternatives and multi-attributes with incomparable units, in which the DMs’ weights play a key role [2, 4, 20]. DMs cannot be supposed to have enough professional knowledge to evaluate all sides of the problem, but rather only specific issues [21]. DMs often have different backgrounds with different expertise, personalities and experiences, which means that the individual DMs are usually not evenly qualified to fairly promote the whole decision process, and may influence the overall decision result [22–24]. That is, the DMs’ weights may be different. However, DMs’ weights are usually ignored in the MAGDM literature [24]. Most of the existing studies often suppose that the weights of the DMs are known or not taken it into account, and this increases the irrationality in the process [23]. Hence, how to obtain the weights of DMs in MAGDM is an important and interesting research topic [3, 22, 25].

Many approaches have been proposed to obtain DMs’ weights. For example, Bodily [26] presented a decision-making term to the initial DMs through measuring the extra preference value deviations. Brock [27] proposed a Nash bargaining-based method to obtain DMs’ weights inherently. Ramanathan and Ganesh [21] used the analytic hierarchy process (AHP) approach to obtain the DMs’ weights, in which each expert gives their evaluation opinions to other experts. Eklund [28] used a consensus approach to obtain the DMs’ weights. DMs may adjust their assessment opinions if suggested by a chairman. Xu [29] improved Bodily’s approach to calculate the DMs’ weights. Xu [30] also proposed some particular formulas to compute the DMs’ weights. Parreiras et al. [31] established a consensus model to obtain the DMs’ weights. Wan and Xu [32] presented two approaches to gain the weights of DMs according to the similarity degree. Zhang and Xu [33] established a goal-programming approach to obtain the DMs’ weights. Pérez and Cabrerizo [34] proposed a consensus approach to get the weights of DMs. Yue [3] proposed a novel approach based on Technique for Order Preference by Similarity to an Ideal Solution (TOPSIS) and entropy to gain the weights of DMs. Liang et al.[20] presented a prospect theory-based method to calculate the weights of DMs in GDM, in which DMs’ weights can be represented using interval numbers, exact numbers and rankings.

In these studies of determining DMs’ weights in MAGDM problems, Yue did more in-depth research with objective and subjective evaluation information with real numbers, interval numbers, intuitionistic fuzzy sets (IFSs), interval-valued intuitionistic fuzzy sets (IVIFSs), and other. For instance, Yue [22] determined DMs’ weights with crisp values based on an improved TOPSIS. In the same year, Yue also extended TOPSIS for determining DMs’ weights in MAGDM with interval numbers [35] and IVIFS [36]. Yue also proposed a projection method [2] and a straightforward approach [24] for the determination of the DMs’ weights with interval data. Yue [37] extended the original TOPSIS to obtain the DMs’ weights with uncertain information. Yue [38] presented an extended TOPSIS, which was used twice in MAGDM with multi-attribute interval data; the proposed method was first used to obtain the DMs’ weights and, second, to sort the order of the alternatives. Yue [39] also presented an extended TOPSIS method with a systematic methodology that can obtain the DMs’ weights without information loss. Yue [40] combined TOPSIS and the optimistic coefficient to determine the DMs’ weights in MAGDM under the IVIFS environment. Yue [41] improved the extended TOPSIS technique to obtain DMs’ weights for MAGDM in IFSs.

The abovementioned studies have contributed substantially to obtaining DMs’ weights under MAGDM and can be divided into two categories [6, 23, 24]. (i) Subjective weighting, It occurs on the basis of DMs’ subjective comments. The DMs’ weights are often offered ahead of schedule or by the contrast of differences among DMs via a particular assessment matrix (e.g., Delphi, AHP) [21, 28]. In this way, the DMs should comprehend each other, but even then, the subjectivity and uncertainty is still high. Therefore, scholars have not further researched this area. (ii) Objective weighting. It occurs just by information offered in each alternative’s decision matrix. In these studies [29–31, 39–41], the common aspect is that they do not need to offer another decision matrix for the assessment of DMs, and the DMs’ weights are calculated only by the information provided in the decision matrix for each alternative. Considering the objectives and accuracy provides us with a hot study area.

But, through reviewing the literature, we found that the previous researches on the determination of DMs in MAGDM share something in common, namely, the calculated weight vectors of DMs are generally constant. That is to say, once the weight vector of the DMs is obtained, its quantitative value will not change throughout the entire computation process. For example, although our previous studies [6, 23] used TOPSIS, statistical variance (SV) and simple additive weighting (SAW) to obtain the weights of DMs, the DM weighting method in the approaches are still always constant. Nevertheless, these existing methods sometimes may give unreasonable decision results due to the following reasons. First, because each DM’s knowledge, personal preferences and so on may be quite different from the others, they tend to have a strong personal bias, which results in higher ratings given to the alternatives that correspond to their preferences and lower evaluations to the ones that they dislike. Second, even if the weights of the DMs have been confirmed, they may also appear to be obvious errors due to many “sudden” reasons, such as “he happened to be in a bad mood” or “he was just absent-minded at that time”. In this case, aggregating the evaluation information will lead to an unfair or unreasonable decision result. To solve the problem, people often remove the highest and lowest evaluation values for each alternative and then calculate the final decision result. Although this method is simple and feasible, it cannot completely solve the problem. For example, assume there are 30 DMs. Three DMs give significantly higher grades than the others for an alternative, and two DMs give significantly lower grades. If we remove the highest and lowest evaluation values, we cannot eliminate two unreasonable evaluation opinions, and the other unreasonable evaluation opinion(s) still can affect the decision result. Therefore, always treating the weight vectors of the DMs as constant in the decision process, especially those decision problems that require repeated evaluations (e.g., singing contest scores), may increase the irrationality of the final decision result.

Therefore, from the above analysis, we can see that neither the subjective or objective weighting method can handle the problem very well. Therefore, to solve the above situation, we propose a novel approach to obtain the DMs’ weights for intuitionistic fuzzy group decision making based on the variable weights theory [42, 43]. In our approach, the computed weights of DMs are not constant, but rather they change according to the needs of higher management or the actual situation. This makes the determination of DMs’ weights more accurate and eliminates the influence of unreasonable evaluation opinions on the final decision.

Our method is composed of the following main steps. First, DMs provide their assessment information based on each attribute with IFSs and compute the weighted decision matrix according to the intuitionistic fuzzy weighted averaging (IFWA) operator with the prior given attributes and DMs’ weights. Second, the determination of the DMs’ weights is based on the variable weighting method, and the conversion of individual decisions to alternative decisions. Third, the alternatives are ranked.

The article is organized as follows. Section 2 presents the basic concepts of IFSs and variable weights theory. Based on an extended variable weight vector, Section 3 develops a MAGDM methodology with intuitionistic fuzzy numbers that can determine the DMs’ weights. Section 4 gives a comparison between our method and other studies. Section 5 presents two illustrative examples, and Section 6 concludes the article.

## 2 Preliminaries

In this segment, we will review the basic concepts related to IFSs and variable weights theory.

### 2.1 Intuitionistic fuzzy sets

Let *A* be an intuitionistic fuzzy (IF) set in the universe of discourse *X*, where *A* = {〈*x*_*i*_,*μ*_*A*_(*x*_*i*_),*v*_*A*_(*x*_*i*_)〉|*x*_*i*_∈*X*}, and *μ*_*A*_ and *v*_*A*_ are the membership function and the non-membership function of the membership degree and the non-membership degree of an element, respectively. *x*_*i*_ belongs to the IF set *A*, where *μ*_*A*_(*x*_*i*_)∈[0,1], *v*_*A*_(*x*_*i*_)∈[0,1], 0≤*μ*_*A*_(*x*_*i*_)+*v*_*A*_(*x*_*i*_)≤1 and 1≤*i*≤*m*. The degree of indeterminacy *π*_*A*_(*x*_*i*_) of element *x*_*i*_ belonging to the IF set *A* is equal to 1−*μ*_*A*_(*x*_*i*_)+*v*_*A*_(*x*_*i*_), where *π*_*A*_(*x*_*i*_)∈[0,1] and 1≤*i*≤*m*. According to [44], the IF value of element *x*_*i*_ belonging to the IF set *A* is represented by (*μ*_*A*_(*x*_*i*_),*v*_*A*_(*x*_*i*_)), where 1≤*i*≤*m*.

**Definition 1**[44]: Let *A* and *B* be two IFSs given as *A* = {〈*x*,*μ*_*A*_(*x*_*i*_),*v*_*A*_(*x*_*i*_)〉|*x*_*i*_∈*X*} and *B* = {〈*x*_*i*_,*μ*_*B*_(*x*_*i*_),*v*_*B*_(*x*_*i*_)〉|*x*_*i*_∈*X*}, where

$$\begin{array}{l} A\subseteq B\Leftrightarrow \left(\forall x_{i}\in X\right)\mu_{A}\left(x_{i}\right)\leq \mu_{B}\left(x_{i}\right)\&v_{A}\left(x_{i}\right)\geq v_{B}(x_{i}),\\ A=B\Leftrightarrow \left(\forall x_{i}\in X\right)\mu_{A}\left(x_{i}\right)=\mu_{B}\left(x_{i}\right)\&v_{A}\left(x_{i}\right)=v_{B}(x_{i}),\\ A\cup B\Leftrightarrow \{\langle x_{i},\mu_{A}\left(x_{i}\right)\cup \mu_{B}\left(x_{i}\right),v_{A}\left(x_{i}\right)\cap v_{B}\left(x_{i}\right)\rangle |x_{i}\in X\},\\ A\cap B\Leftrightarrow \{\langle x,\mu_{A}\left(x\right)\cap \mu_{B}\left(x\right),v_{A}\left(x\right)\cup v_{B}\left(x\right)\rangle |x\in X\},\;\mathrm{and}\\ A^{n}=\left\{\langle x_{i},{\left[\mu_{A}\left(x_{i}\right)\right]}^{2},{\left[v_{A}\left(x_{i}\right)\right]}^{2}\rangle |x_{i}\in X\right\}. \end{array}$$

Let *A* and *B* be two IFSs given as *A* = {*μ*_*A*_(*x*_*i*_),*v*_*A*_(*x*_*i*_)} and *B* = {*μ*_*B*_(*x*_*i*_),*v*_*B*_(*x*_*i*_)}, where *k* is a real number greater than 0:

$$A+B=\left\{\mu_{A}\left(x_{i}\right)+\mu_{B}\left(x_{i}\right)-\mu_{A}\left(x_{i}\right)*\mu_{B}\left(x_{i}\right),v_{A}\left(x_{i}\right)*v_{B}\left(x_{i}\right)\right\}$$ (1)

$$kA=\left\{1-\left(1-\mu_{A}{\left(x_{i}\right)}^{k}\right),v_{A}{\left(x_{i}\right)}^{k}\right\}$$ (2)

$$d\left(A,B\right)=\frac{1}{2}\left(\left|\mu_{A}\left(x_{i}\right)-\mu_{B}\left(x_{i}\right)\right|+\left|v_{A}\left(x_{i}\right)-v_{B}\left(x_{i}\right)\right|+\left|\mu_{B}\left(x_{i}\right)+v_{B}\left(x_{i}\right)-\left(\mu_{A}\left(x_{i}\right)-v_{A}\left(x_{i}\right)\right)\right|\right)$$ (3)

Xu et al. [45] and Xu [46] gave a procedure for ranking IFVs, which can be defined as follows.

**Definition 2**[12, 45]: Let *A* = {*μ*_*A*_(*x*_*i*_),*v*_*A*_(*x*_*i*_)} and *B* = {*μ*_*B*_(*x*_*i*_),*v*_*B*_(*x*_*i*_)} be two IFVs; *S*(*A*) = *μ*_*A*_(*x*_*i*_)−*v*_*A*_(*x*_*i*_) and *S*(*B*) = *μ*_*B*_(*x*_*i*_)−*v*_*B*_(*x*_*i*_) be the scores of *A* and *B*, respectively; and *H*(*A*) = *μ*_*A*_(*x*_*i*_)+*v*_*A*_(*x*_*i*_) and *H*(*B*) = *μ*_*B*_(*x*_*i*_)+*v*_*B*_(*x*_*i*_) be the accuracy degrees (AD) of *A* and *B*. Then,

(1) if *S*(*A*)<*S*(*B*), then *A* is smaller than *B*, which is denoted as *A*<*B*;

(2) if *S*(*A*) = *S*(*B*), then

(a) if *H*(*A*) = *H*(*B*), then *A* and *B* represent the same information, i.e., *μ*_*B*_(*x*_*i*_) = *μ*_*A*_(*x*_*i*_) and *v*_*B*_(*x*_*i*_) = *v*_*A*_(*x*_*i*_), which is denoted as *A* = *B*; and

(b) if *H*(*A*)<*H*(*B*), then *A* is smaller than *B*, which is denoted as *A*<*B*.

### 2.2 Variable weights

The variable weights theory was proposed by Li [43] and Zhang et al. [42]. The basic concept is as follows [43].

A common multifactor function is the mapping (operator), that is applied in additional decision-making systems and is as follows:

$$M_{t}\left(x_{1},x_{2},\ldots x_{t}\right)=\sum \left(x_{1},x_{2},\ldots x_{t}\right)\overset{\Delta }{=}\sum_{k=1}^{t}\lambda_{k}x_{k}$$ (4)

where *λ*_*k*_∈[0,1](*k* = 1,2,…*m*) refer to the weighted average or weighted mean, and $\sum_{k=1}^{t}\lambda_{k}=1$. Due to the weights {*λ*_*k*_} are constant, the formula represents a constant synthetic weight on the situation {*x*_*k*_} with respect to relevant factors. Let *λ* = (*λ*_1_,*λ*_2_,…,*λ*_*t*_). Afterwards, the *λ* is defined as the constant weight vector. The constants reflect the strength or relative importance of the correlative factors.

Refer to the constant weight, the weight vector *λ* is always fixed, in spite of the essential structure or configuration of the objective function *ϑ*(*f*(*u*)) = (*ϑ*_1_(*f*_1_(*u*)),*ϑ*_2_(*f*_2_(*u*)),…,*ϑ*_*t*_(*f*_*t*_(*u*))).

The “structure or configuration” denotes the function relationship of *u* and the vector *ϑ*(*f*(*u*))’s elements. The constant vector *λ* in **Eq (4)** with no changes when *X* = (*x*_1_,*x*_2_,…,*x*_*t*_) varies. Hence, the application of constant weights has its limitations. Li [43] gave an example to illustrate that the constant vector *λ* is unfit to apply in all values of *X*.

**Example 1**[43]. Assume there are two factors, *x*_1_ = feasibility and *x*_2_ = necessity, to evaluate an engineering system. Let the two factors are equally important, i.e.,

$$\lambda =\left(\lambda_{1},\lambda_{2}\right)=\left(0.5,0.5\right)$$

Hence, on the basis of **Eq (4)**, the follows are the multifactorial function:

$$M_{2}\left(x_{1},x_{2}\right)=\sum \left(x_{1},x_{2}\right)=0.5x_{1}+0.5x_{2}$$

There are two scenarios to consider:

1. the project is entirely viable, but it has very low inevitability; and
2. the project maybe in great need, yet it is not viable.

Generally, we disagree with the project regardless of the circumstances because of the quite low value of the project.

Numerically, let *X* = (0.1,0.9)∈*X*(*f*_1_)×*X*(*f*_2_) and X* = (0.5,0.5)∈*X*(*f*_1_)×*X*(*f*_2_). Afterwards, we would expect *M*_2_(*X*)<<*M*_2_(X*) in most cases. However, if we use the constant weight vector, we have

$$M_{2}\left(x_{1},x_{2}\right)=0.5x_{1}+0.5x_{2}$$

This consequence contradicts ordinary perceptions.

To overcome these disadvantages in the constant weights method, Li [43] and Zhang et al. [42] put forward the idea of the variable weights theory. The axioms and definition of the variable weights are as below.

**Definition 3** [42, 43]: A set of n-dimensional variable weights with a penalty is a set of *t*-ary mappings

*λ*_*k*_:[0,1]^*t*^→[0,1]^*t*^

(*x*_1_,*x*_2_,…,*x*_*t*_)↦*λ*_*k*_(*x*_1_,*x*_2_,…,*x*_*t*_) that meet the below axioms:

*λ.1). NORMALITY.* $\sum_{k=1}^{t}\lambda_{k}\left(x_{1},x_{2},\ldots ,x_{t}\right)=1$;

*λ*.*2)*. *CONTINUITY*. *λ*_*k*_(*k* = 1,2,…,*t*) is continuous in the *t*-dimensional space; and

*λ*.*3)*. *PENALTY*. *λ*_*k*_(*x*_1_,*x*_2_,…,*x*_*t*_) is monotonically decreasing according to *x*_*k*_(*k* = 1,2,..,*t*).

Let *λ*(*x*) = (*λ*_1_(*x*),(*λ*_2_(*x*),…,*λ*_*t*_(x)), and we define it with the variable weight vector with a penalty. If we change *λ*.*3)* to meet the condition that *λ*_*k*_(*x*_1_,*x*_2_,…,*x*_*t*_) is monotonically increasing according to *x*_*k*_(*k* = 1,2,..,*t*), we define it as the variable weight vector with a reward.

To capture the varying rule of weights, the axiomatic definition of the state variable weight vector was modified in the literature as follows.

**Definition 4:** A mapping

$$S:{\left[0,1\right]}^{t}\to {\left[0,1\right]}^{t}$$

$$X\to S\left(X\right)=\left(S_{1}\left(X\right),\ldots ,S_{t}\left(X\right)\right)$$

where *S*_*k*_:[0,1]^*t*^→[0,1]^*t*^ and X→S_*k*_(*X*) is defined as an (*t*-dimensional) state variable weight vector with a penalty if *S* satisfies the following axioms:

*s*.*1)*. *x*_*a*_≥*x*_*b*_→*S*_*a*_(*X*)≤*S*_*b*_(*X*),

*s*.*2)*. *S*_*a*_(*x*_1_,*x*_2_,…,*x*_*t*_) is continuous according to each variable *x*_*b*_(*a*,*b* = 1,2,…,*t*), and

*s*.*3)*. it satisfies the variable weight vector acquired from Eq (5) as

$$\lambda \left(x\right)=\frac{\lambda *S\left(X\right)}{\sum_{k=1}^{t}\lambda_{k}S_{k}\left(X\right)}$$ (5)

Eq (5) satisfies axioms *λ*.*1) -λ*.*3)* in **Definition 3**, where *λ* = (*λ*_1_,*λ*_2_,…,*λ*_*t*_) is a constant weight vector and λ*S(*X*) = (*λ*_1_*S*_1_(*X*),…,*λ*_t_*S_*t*_(*X*)).

If we change *s*.*1)* to meet the conditions that *x*_*a*_≥*x*_*b*_→*S*_*a*_(*X*)≥*S*_*b*_(*X*) and *λ*_*k*_(*x*_1_,*x*_2_,…,*x*_*t*_) is monotonically increasing according to *x*_*k*_(*k* = 1,2,…,*t*), we call it the state variable weight vector with a reward.

## 3. The proposed method

**Fig 2:**
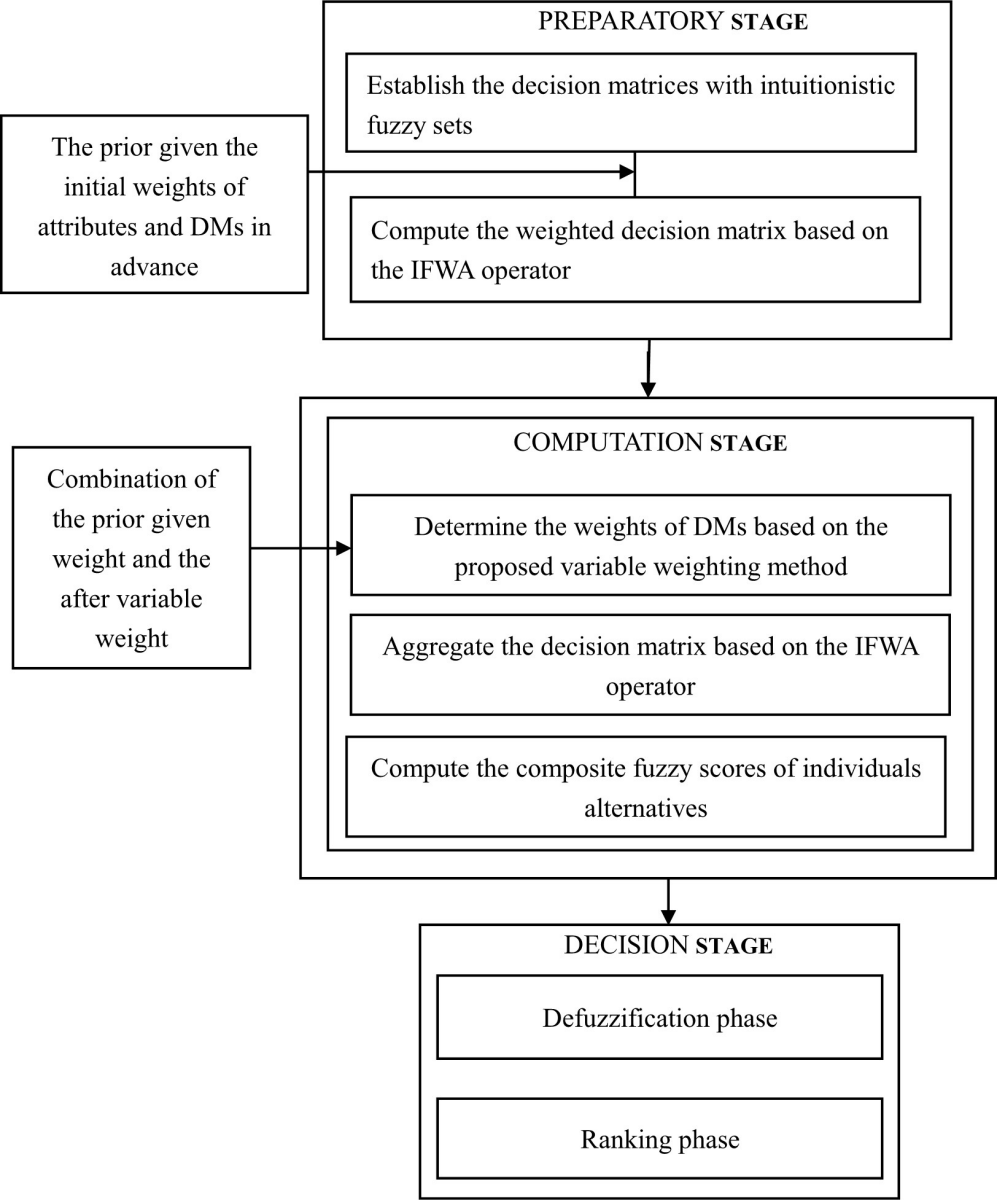
The conceptual framework of the proposed method.

The proposed approach performs the below three steps: (1) Preparatory stage. In the preparatory stage, DMs present their assessment information based on each attribute using intuitionistic fuzzy numbers and establish the decision matrix. Then, we compute the weighted decision matrix based on the IFWA operator with the prior given attributes and DMs’ weights. (2) Computation stage. In the computation stage, a variable weighting approach is proposed to obtain the DMs’ weights. After that, we aggregate the decision matrix based on the IFWA operator and work out the composite fuzzy scores of individual alternatives. (3) Decision stage. In the end, in the decision stage, the most desired alternatives are then selected with the crisp values of composite scores, which are calculated by the accuracy degrees operator (ADO) of IFSs from **Definition 2**. Fig 2 represents the computational process of the proposed approach.

### 3.1 Notations and definitions

Let *M* = {1,2,…,*m*}, *N* = {1,2,…,*n*} and *T* = {1,2,…,*t*}, where *i*∈*M*,*j*∈*N*,*k*∈*T*. Let *A* = {*A*_1_,*A*_2_,…,*A*_*m*_} (*m*≥2) be a discrete set of m feasible alternatives, *U* = {*u*_1_,*u*_2_,…,*u*_*n*_} be a finite set of attributes, and *D* = {*d*_1_,*d*_2_,…,*d*_*t*_} be a group of DMs.

Specifically, the following notations are defined:

*M*: *M* = {1,2,…,*m*}, where *i*∈*M*;

*N*: *N* = {1,2,…,*n*}, where *j*∈*N*;

*T*: *T* = {1,2,…,*t*}, where *k*∈*T*;

*A*: *A* = {*A*_1_,*A*_2_,…,*A*_*m*_} (*m*≥2) denotes a discrete set of *m* feasible alternatives;

*U*: *U* = {*u*_1_,*u*_2_,…,*u*_*n*_} denotes a finite set of attributes;

*D*: *D* = {*d*_1_,*d*_2_,…,*d*_*t*_} denotes a group of DMs;

*λ*_*k*_: *λ*_*k*_*(k=1*,*2*,*…*,*t)* denotes the initial DMs’ weights;

*q*: *q* = (*q*_1_,*q*_2_,…,*q*_*t*_) denotes the accuracy degrees;

*λ*(*q*): *λ*(*q*) = (*λ*_1_(*q*),*λ*_2_(*q*),…,*λ*_*t*_(*q*)) denotes the variable weight vector;

### 3.2 Collection of assessment information and construction of the decision matrices

An MAGDM with IFSs can be described as follows.

Let

$$X_{k}={\left(x_{ij}^{k}\right)}_{m\times n}=\begin{array}{c} A_{1}\\ A_{2}\\ \vdots \\ A_{m} \end{array}\left[\begin{array}{cccc} u_{1} & u_{2} & \cdots & u_{n}\\ \left(\mu_{11}^{k},v_{11}^{k}\right) & \left(\mu_{12}^{k},v_{12}^{k}\right) & \cdots & \left(\mu_{1n}^{k},v_{1n}^{k}\right)\\ \left(\mu_{21}^{k},v_{21}^{k}\right) & \left(\mu_{22}^{k},v_{22}^{k}\right) & \cdots & \left(\mu_{2n}^{k},v_{2n}^{k}\right)\\ \vdots & \vdots & \vdots & \vdots \\ \left(\mu_{m1}^{k},v_{m1}^{k}\right) & \left(\mu_{m2}^{k},v_{m2}^{k}\right) & \cdots & \left(\mu_{mn}^{k},v_{mn}^{k}\right) \end{array}\right]$$ (6)

In the field of MAGDM research, many scholars have realized that DMs’ weights play a key role in MAGDM problems [23, 38, 41]. Therefore, we focus on how to determine the DMs’ weights.

First, we need to compute the weighted decision matrix $x_{i}^{k}$ based on the IFWA operator [46]:

$$IFWA\left(x_{i1}^{k},x_{i2}^{k},\ldots ,x_{in}^{k}\right)=\overset{n}{\underset{j=1}{⊕}}x_{ij}^{k}w_{j}^{}=\left(1-\prod_{j=1}^{n}{\left(1-\mu_{ij}^{k}\right)}^{w_{j}^{}},\prod_{j=1}^{n}{\left(v_{ij}^{k}\right)}^{w_{j}^{}}\right)$$ (7)

where *w*_*1*_, *w*_*2*_,…, and *w*_*j*_ are the given attribute weights, and they meet the conditions $\sum_{j=1}^{n}w_{j}^{}=1$ and *w*_*j*_∈[0,1]

**Example 2.** Let

$$\begin{array}{l} D_{1}=\begin{array}{c} A_{1}\\ A_{2}\\ A_{3} \end{array}\left[\begin{array}{ccc} u_{1} & u_{2} & u_{3}\\ \left(0.6,0.3\right) & \left(0.1,0.5\right) & \left(0.3,0.4\right)\\ \left(0.4,0.1\right) & \left(0.6,0.1\right) & \left(0.2,0.3\right)\\ \left(0.1,0.5\right) & \left(0.5,0.3\right) & \left(0.6,0.2\right) \end{array}\right],D_{2}=\begin{array}{c} A_{1}\\ A_{2}\\ A_{3} \end{array}[\begin{array}{ccc} u_{1} & u_{2} & u_{3}\\ \left(0.5,0.4\right) & \left(0.6,0.3\right) & \left(0.4,0.6\right)\\ \left(0.7,0.4\right) & \left(0.3,0.6\right) & \left(0.4,0.5\right)\\ \left(0.8,0.1\right) & \left(0.4,0.5\right) & \left(0.3,0.3\right) \end{array}],\\ D_{3}=\begin{array}{c} A_{1}\\ A_{2}\\ A_{3} \end{array}\left[\begin{array}{ccc} u_{1} & u_{2} & u_{3}\\ \left(0.3,0.6\right) & \left(0.1,0.5\right) & \left(0.5,0.4\right)\\ \left(0.6,0.3\right) & \left(0.4,0.2\right) & \left(0.5,0.1\right)\\ \left(0.3,0.4\right) & \left(0.3,0.4\right) & \left(0.5,0.4\right) \end{array}\right],\;\mathrm{and}\;w=\left[\begin{array}{ccc} 0.35 & 0.34 & 0.31 \end{array}\right] \end{array}$$

be three intuitionistic fuzzy sets and the attribute weights. Then, $x_{3}^{3}$ can be calculated by Eq (7) as follows:

$$x_{3}^{3}=\begin{array}{c} A_{1}\\ A_{2}\\ A_{3} \end{array}\left[\begin{array}{ccc} d_{1} & d_{2} & d_{3}\\ \left(0.372,0.390\right) & \left(0.510,0.411\right) & \left(0.313,0.497\right)\\ \left(0.429,0.141\right) & \left(0.504,0.492\right) & \left(0.508,0.186\right)\\ \left(0.427,0.316\right) & \left(0.572,0.243\right) & \left(0.369,0.400\right) \end{array}\right]$$

### 3.3 Obtaining the DMs’ weights based on variable weights theory

First, let *λ*_*k*_*(k=1*,*2*,*…*,*t)* denote the prior DMs’ weights based on the opinions from interviewing the senior DM. We called it the initial DMs’ weights, which meet $\sum_{k=1}^{t}\lambda_{k}=1$ and *λ*_*k*_∈[0,1].

Then, let *q*(*q*_1_,*q*_2_,…,*q*_*t*_) be the accuracy degrees of $x_{i}^{k}$, which are calculated by **Definition 2**, where *q*_*k*_∈[0,1]^*l*^. In addition, let *λ*(*q*) = (*λ*_1_(*q*),*λ*_2_(*q*),…,*λ*_*t*_(*q*)) be the variable weight vector shown as follows:

$$\lambda_{k}\left(q\right)=\lambda_{k}+\gamma \lambda_{k}\left(\sum_{k=1}^{t}(\mu_{}^{k}+v_{}^{k})\lambda_{k}-(\mu_{}^{k}+v_{}^{k})\right).$$ (8)

where *k* = 1,2,…,*t*; $\bar{q}=\sum_{k=1}^{t}(\mu_{}^{k}+v_{}^{k})\lambda_{k}$; *q*_*k*_ = (*μ*^*k*^+*v*^*k*^) and parameter *γ* satisfy:

$$\frac{1}{\underset{q\in {\left[0,1\right]}_{}^{t}}{\min}\;\underset{1\leq k\leq t}{\min}\left(q_{k}-\bar{q}\right)}\leq \gamma <\frac{1}{\underset{q\in {\left[0,1\right]}_{}^{t}}{\max}\;\underset{1\leq k\leq t}{\max}\left(q_{k}-\bar{q}\right)}$$ (9)

**Theorem 1:** *λ*(*q*) is a variable weight vector. When *γ*>0, *λ*(*q*) is the variable weight vector with a penalty; when *γ*<0, *λ*(*q*) is variable weight vector with a reward.

**Proof**.

Obviously, *λ*(*q*) satisfies the continuity in **Definition 3**. If

$$0\leq \gamma \leq \frac{1}{\begin{array}{cc} \begin{array}{c} \max\\ q\in {\left[0,1\right]}^{t} \end{array} & \begin{array}{c} \max\\ 1\leq k\leq t \end{array}\left(q_{k}-\bar{q}\right) \end{array}}.$$

for *q*∈[0,1]^*t*^, when $\left(\bar{q}-q_{k}\right)\geq 0$, we can obtain $\lambda_{k}\left(q\right)=\left(1+\gamma \left(\bar{q}-q_{k}\right)\right)\lambda_{k}\geq 0$. When $\left(\bar{q}-q_{k}\right)<0$, it gives

$$\lambda_{k}\left(q\right)=\left(1+\gamma \left(\bar{q}-q_{k}\right)\right)\lambda_{k}\geq \left(1+\frac{\bar{q}-q_{k}}{\begin{array}{cc} \begin{array}{c} \max\\ q\in {\left[0,1\right]}^{t} \end{array} & \begin{array}{c} \max\\ 1\leq k\leq t \end{array}\left(q_{k}-\bar{q}\right) \end{array}}\right)\lambda_{k}\geq \left(1+\frac{\bar{q}-q_{k}}{q_{k}-\bar{q}}\right)\lambda_{k}=0.$$

Because $\sum_{k=1}^{t}\lambda_{k}\left(q\right)=\sum_{k=1}^{t}\left(1+\gamma \left(\bar{q}-q_{k}\right)\right)\lambda_{k}=\sum_{k=1}^{t}\lambda_{k}+\gamma \left(\bar{q}\sum_{k=1}^{t}\lambda_{k}-\sum_{k=1}^{t}q_{k}\lambda_{k}\right)=1$, *λ*(*q*) satisfies the normality in **Definition 3.**

Because *γλ*_*k*_(*q*)/*γλ*_*k*_ = *γ*(*q*_*k*_−1)<0, *λ*_*k*_(*q*) is monotonically decreasing with variable *q*_*k*_. That is, *λ*(*q*) is a variable weight vector with a penalty.

In addition, $\frac{\lambda_{a}\left(q\right)}{\lambda_{a}}=1+\gamma \sum_{a\neq b}^{}q_{b}\lambda_{b}+\gamma \left(\lambda_{a}-1\right)q_{b}$ is clearly monotonically decreasing about *q*_*b*_ and thus meets **Definition 4**.

Let

$$S_{k}(q)=\frac{1+\gamma \left(\bar{q}-q_{k}\right)}{t\left(1+\gamma \bar{q}\right)-\sum_{k=1}^{t}q_{k}},k=1,2,\ldots t$$ (10)

Based on **Definition 4**, *S*(*q*) =(*S*_1_(*q*),*S*_2_(*q*),…*S*_*t*_(*q*)) is the penalty variable weight vector of *λ*(*q*).

The same theory proves that when

$$\frac{1}{\begin{array}{cc} \begin{array}{c} \min\\ q\in {\left[0,1\right]}_{}^{t} \end{array} & \begin{array}{c} \min\\ 1\leq k\leq t \end{array}\left(q_{k}-\bar{q}\right) \end{array}}\leq \gamma <0,$$

*λ*(*q*) is a reward variable weight vector, which is defined as **Definition 3**, and *S*(*q*) is also a state variable weights vector with a reward because its form is the same as Eq (10).

According to **Theorem 1**, when *γ*<0, *λ*(*q*) is a variable weight vector with a penalty; when *γ*>0, *λ*(*q*) is a variable weight vector with a reward; and when *γ* = 0, *λ*(*q*) = *λ* is the initial given weight. This reflects that we can obtain diverse variable weight vectors when we regulate the parameters accordingly, which provides a great convenience in practical use.

### 3.4 Computing the comprehensive evaluation value

After the DMs’ weights are computed, then we can compute the comprehensive evaluation value based on the IFWA operator [46], using (11) as follows:

$$IFWA\left(x_{i}^{1},x_{i}^{2},\ldots ,x_{i}^{t}\right)=\overset{t}{\underset{k=1}{⊕}}x_{i}^{k}\lambda_{k}\left(q\right)=\left(1-\prod_{k=1}^{t}{\left(1-\mu_{i}^{k}\right)}^{\lambda_{k}\left(q\right)},\prod_{k=1}^{t}{\left(v_{i}^{k}\right)}^{\lambda_{k}\left(q\right)}\right)$$ (11)

Now, we can sort the alternatives *A*_*i*_(*i*∈*m*) according to *S*_*i*_(*i*∈*m*) in descending order according to **Definition 2**.

$$S_{i}=\mu_{i}-v_{i}$$ (12)

### 3.5 Main steps of the proposed method

The primary steps of the proposed method are summarized as follows:

**Step 1.** Construct the individual decision matrix ${\left(x_{ij}^{k}\right)}_{m\times n}$ in groups by Eq (6),

**Step 2.** Compute the weighted normalized decision matrix $x_{i}^{k}$ by Eq (7) with the prior given attributes and the DMs’ weight vectors $w_{j}^{k}$ and *λ*_*k*_,

**Step 3.** Determine the DMs’ weights by using Eqs (8–10),

**Step 4.** Compute the collective group decision by Eq (11) with the obtained weight vector *λ*_*k*_(*q*) of the DMs, and

**Step 5.** Rank all the alternatives according to the value of *S*_*i*_ by using Eq (12).

## 4. Comparison between the existing related approaches

As mentioned in the introduction section, many studies researched the acquisition of the DMs’ weights in MAGDM problems with real numbers, interval numbers, IFSs, IVIFs, and others [23, 29, 39–41]. The existing methods can be divided into two categories [23, 24]: (i) Subjective weighting, which occurs on the basis of the DMs’ subjective comments; and (ii) Objective weighting, which occurs on the basis of the information that is offered in the decision matrix for each alternative for certain attributes.

In the literature review on the acquisition of DMs’ weights over the past decades, the objective weighting approaches gained more attention in comparison to subjective weighting methods. With regard to objective weighting methods, Yue et al. did more in-depth research on this problem [38, 40, 41]. They developed a TOPSIS approach to obtain the DMs’ weights and define the positive ideal solution as the average of the evaluation information. The negative ideal solution included two aspects, the right and left negative ideal solutions, which are the maximum and minimum matrices of the group decision information, respectively. Then, they combined the approach with other methods (e.g., projection, straightforward, optimistic coefficient, etc.) under MAGDM with real numbers, interval numbers, IFs, IVIFs, and others [24, 36, 39]. However, in Yue’s approach, once the weight vector of the DMs is computed, its numerical value will no longer change. As mentioned in the introduction, always treating the weight vector of DMs as a constant value may cause the end decision result to be irrational, especially in those decision problems requiring repeated evaluations (e.g., singing contest scores).

Compared with the subjective weighting methods [21, 28], our method is more objective (the computation of the variable weight in our method is based on the decision matrix of the alternatives) and it does not lose the subjective opinions (the prior given DMs’ weights are based on subjective preferences). Therefore, our method is to some extent more accurate than the subjective weighting approaches. Table 1 represents the comparisons between our method and two kinds of existing related approaches.

**Table 1:** Comparisons between our approach and the two related approaches.

Characteristics	Objective weighting methods	Subjective weighting methods	This paper
	Proposed by [2, 22, 24, 35–41]	Proposed by [21, 28]	
Decision information	Interval numbers, intuitionistic fuzzy sets (IFSs) and interval-valued intuitionistic fuzzy sets (IVIFSs)	Crisp values	Intuitionistic fuzzy sets (IFSs)
Weight of DM	Derived from individual decisions	A priori given	Derived from the combination of the a priori and a posteriori variable weight vectors
Whether the Weight of the DM is variable or constant	Constant	Constant	Variable
Decision results	Ranking the order of alternatives	Ranking the order of alternatives	Ranking the order of alternatives

**Fig 3:**
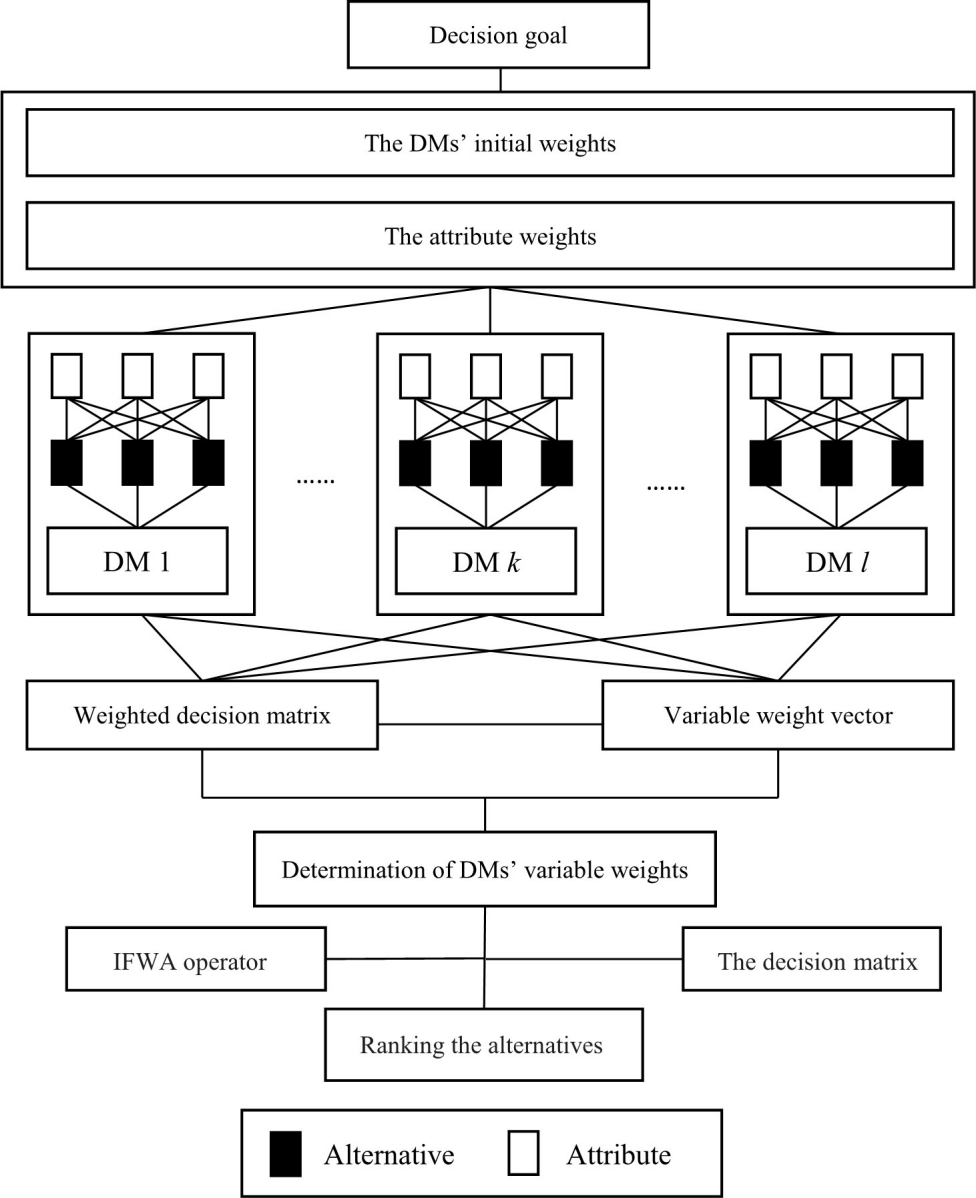
Hierarchical structure of the proposed method.

The primary difference between the proposed approach and other approaches is that the DMs’ weights are not constant in the decision process once they are calculated, but they change according to the needs of top management or the actual situation. In the proposed approach, the weight vector of DMs is defined as a variable weight vector. In addition, we add adjustable parameters to the variable weight vector to measure the capability of changing weights. Compared with existing methods, our method can reflect the preferences of top management to the DMs and give adjustment space for aggregating the evaluation information when some DMs give obviously unreasonable opinions. Fig 3 illustrates the hierarchical structure of our method.

## 5. Illustrative examples

Two numerical examples are applied to compare and discuss the decision results of the proposed method computed in the methods presented in Yue [41]’s and Chen et al [47]’s studies.

Example 1: adapted from Yue [41]:

An annual report is needed to assess satisfaction in regard to leadership at Chinese universities. Let the alternatives *A*_*1*_, *A*_*2*_, *A*_*3*_, and *A*_*4*_ be four leaders of a university in Guangdong, China, namely, the headmaster and three vice-headmasters, whose opinions would be assessed. There are three groups of the various staff, including teachers (*d*_*1*_), scholars (*d*_*2*_) and students (*d*_*3*_), who are the DMs (evaluators) for the grading. There are three attributes *u*_*1*_, *u*_*2*_ and *u*_*3*_ that are used to evaluate the alternatives *A*_*1*_, *A*_*2*_, *A*_*3*_, and *A*_*4*_, as shown below:

1. *u*_*1*_=working experience,
2. *u*_*2*_=academic performance, and
3. *u*_*3*_=personality.

Suppose that the decision matrices *X*_*1*_, *X*_*2*_ and *X*_*3*_ are represented by IFs given by the decision makers *d*_*1*_, *d*_*2*_ and *d*_*3*_, respectively and are shown in Table 2.

**Table 2:** Three score attribute matrices.

Experts’	Alternatives	Attribute		
assessment		*u*_*1*_	*u*_*2*_	*u*_*3*_
*d*_*1*_	*A*_*1*_	(0.36,0,27)	(0.53,0.28)	(0.57,0.22)
	*A*_*2*_	(0.72,0.28)	(0.91,0.07)	(0.80,0.10)
	*A*_*3*_	(0.63,0.19)	(0.88,0.12)	(0.86,0.14)
	*A*_*4*_	(0.65,0.33)	(0.72,0.23)	(0.77,0.23)
*d*_*2*_	*A*_*1*_	(0.53,0.26)	(0.54,0.35)	(0.68,0.32)
	*A*_*2*_	(0.85,0.15)	(0.86,0.13)	(0.69,0.30)
	*A*_*3*_	(0.83,0.16)	(0.76,0.24)	(0.73,0.13)
	*A*_*4*_	(0.90,0.07)	(0.91,0.03)	(0.66,0.12)
*d*_*3*_	*A*_*1*_	(0.81,0.18)	(0.76,0.24)	(0.74,0.19)
	*A*_*2*_	(0.75,0.16)	(0.84,0.11)	(0.97,0.03)
	*A*_*3*_	(0.89,0.11)	(0.78,0.21)	(0.74,0.11)
	*A*_*4*_	(0.66,0.18)	(0.63,0.27)	(0.71,0.29)

Suppose that the initial weights *λ*_1_, *λ*_2_ and *λ*_3_ of the DMs *d*_*1*_, *d*_*2*_ and *d*_*3*_ are 0.33, 0.34 and 0.33, respectively. Suppose that the attribute weights *u*_*1*_, *u*_*2*_ and *u*_*3*_ given by the DMs *d*_*1*_ are 0.4, 0.2 and 0.4, respectively, i.e., $w_{1}^{1}=0.4$, $w_{2}^{1}=0.2$ and $w_{3}^{1}=0.4$. Suppose that the weights of the attributes *u*_*1*_, *u*_*2*_ and *u*_*3*_ given by the DMs *d*_*2*_ are 0.3, 0.3 and 0.4, respectively, i.e., $w_{1}^{2}=0.3$, $w_{2}^{2}=0.3$ and $w_{3}^{3}=0.4$. Assume that the weights of attributes *u*_*1*_, *u*_*2*_ and *u*_*3*_ given by the DMs *d*_*3*_ are 0.4, 0.4 and 0.2, respectively, i.e., $w_{1}^{3}=0.4$, $w_{2}^{3}=0.4$ and $w_{3}^{3}=0.2$.

**【Step1】:** Based on the decision matrices *d*_*1*_, *d*_*2*_ and *d*_*3*_ and the weights of the attributes given by the DMs, we can obtain the weighted (on attributes) score matrices $x_{i}^{k}$ by Eq (7), as shown in Table 3:

**Table 3:** Three weighted (on attributes) scores matrices.

Alternatives	Decision maker		
	*d*_*1*_	*d*_*2*_	*d*_*3*_
*A*_*1*_	(0.487,0.251)	(0.600,0.309)	(0.778,0.204)
*A*_*2*_	(0.805,0.141)	(0.804,0.190)	(0.863,0.114)
*A*_*3*_	(0.800,0.153)	(0.773,0.166)	(0.828,0.142)
*A*_*4*_	(0.717,0.266)	(0.842,0.067)	(0.659,0.233)

**【Step2】:** Based on Eq (8) and Eq (9), we can obtain the range of *γ* as

−7.20≤γ≤9.46

Assume that *γ* = −7.2, *γ* = 0 and *γ* = 9.46. The reason why we choose the extreme values (-7.2 and 9.46) is that we want to examine the extreme effect of the variable weight in the range of the permit. Then, we can obtain the respective weights of the DMs in different situations, as shown in Table 4.

**Table 4:** The weights of the DMs in different situations.

Different	Alternatives	The weights of the DMs		
parameters		*d*_*1*_	*d*_*2*_	*d*_*3*_
*γ* = −7.2	*A*_*1*_	0.000	0.419	0.581
	*A*_*2*_	0.266	0.391	0.343
	*A*_*3*_	0.328	0.304	0.368
	*A*_*4*_	0.460	0.294	0.245
*γ* = 0	*A*_*1*_	0.330	0.340	0.330
	*A*_*2*_	0.330	0.340	0.330
	*A*_*3*_	0.330	0.340	0.330
	*A*_*4*_	0.330	0.340	0.330
*γ* = 9.46	*A*_*1*_	0.764	0.237	0.000
	*A*_*2*_	0.414	0.273	0.313
	*A*_*3*_	0.333	0.387	0.280
	*A*_*4*_	0.159	0.400	0.441

**【Step3】:** Based on Eq (11), we construct the aggregated decision matrices in different situations, which is shown as Table 5.

**Table 5:** Three individual weighted (on attributes and DMs) decision matrices.

Different	Alternatives	The aggregated decision matrix
parameters		
*γ* = −7.2	*A*_*1*_	(0.716, 0.321)
	*A*_*2*_	(0.827,0.259)
	*A*_*3*_	(0.803,0.244)
	*A*_*4*_	(0.751,0.321)
*γ* = 0	*A*_*1*_	(0.642,0.315)
	*A*_*2*_	(0.826,0.238)
	*A*_*3*_	(0.801,0.259)
	*A*_*4*_	(0.753,0.377)
*γ* = 9.46	*A*_*1*_	(0.516,0.308)
	*A*_*2*_	(0.825,0.212)
	*A*_*3*_	(0.798,0.281)
	*A*_*4*_	(0.757,0.398)

**【Step4】:** Based on Eq (12), we rank all the alternatives in different situations, as shown in Table 6.

**Table 6:** A comparison of the orders of the alternatives of Example 1 for different parameters.

Different parameters	*A*_*1*_	*A*_*2*_	*A*_*3*_	*A*_*4*_
*γ* = −7.2	**3**	**1**	**2**	**4**
*γ* = 0	**4**	**1**	**2**	**3**
*γ* = 9.46	**4**	**1**	**2**	**3**

Table 7 represents a comparison of the ranking order of the alternatives in different approaches.

**Table 7:** A comparison of the orders of the alternatives of Example 1 for different methods.

Methods		Preference order
Xu [48]		*A*_2_>*A*_3_>*A*_4_>*A*_1_
Yue [41]		*A*_2_>*A*_3_>*A*_4_>*A*_1_
Zeng and Su [49]		N/A
Chen et al. [47]		*A*_2_>*A*_3_>*A*_4_>*A*_1_
The proposed method	*γ* = −7.2	*A*_2_>*A*_3_>*A*_1_>*A*_4_
	*γ* =0	*A*_2_>*A*_3_>*A*_4_>*A*_1_
	*γ* = 9.46	*A*_2_>*A*_3_>*A*_4_>*A*_1_

As shown above, with the proposed approach, when *γ* = 0 and *γ* =9.46, Xu [48]’s method, Yue [41]’s method and Chen et al. [47]’s method acquire the same preference order of the alternatives, i.e., *A*_*2*_>*A*_*3*_>*A*_*4*_>*A*_*1*_; however, Zeng and Su [49]’s method cannot handle Example 1 because it does not permit the attributes to have different weights allocated by different DMs. However, we find that the preference order of the alternatives is different (e.g., *A*_*2*_>*A*_*3*_>*A*_*1*_>*A*_*4*_) when *γ* = −7.2. Therefore, the calculation result suggests that through the adjustment of the parameter, top management can adjust the preferences to DMs according to the parameter ∂, and this may affect the final decision result. Apparently, via the above discussion, we can draw a conclusion that the variable weight based weighting approach can to some extent amend the unreasonable sorting results with effect by adjusting the DMs weights, especially when the prior given DMs weights are unconscionable. By adjusting different parameters, the variable weight vector of the DMs also can efficient reflect the differences of each DM’s knowledge, personal preferences and so on, which will lead to more scientific decisions.

Example 2: adapted from Chen et al [47]:

An investment corporation intends to invest a sum of money in the best firm. There are three alternatives firm *A*_*1*_, *A*_*2*_ and *A*_*3*_ to be evaluated as follows:

1. *A*_*1*_: a car firm,
2. *A*_*2*_: a TV firm, and
3. *A*_*3*_: a food firm.

Three attributes *u*_*1*_, *u*_*2*_ and *u*_*3*_ are to be used to evaluate the three alternatives *A*_*1*_, *A*_*2*_ and *A*_*3*_ by the three DMs, i.e., the director (*d*_*1*_), the manager (*d*_*2*_) and the assistant manager (*d*_*3*_), as shown below:

1. *u*_*1*_: the risk criterion,
2. *u*_*2*_: the growth criterion, and
3. *u*_*3*_: the environmental impact criterion.

The decision matrices *X*_*1*_, *X*_*2*_ and *X*_*3*_ given by the decision makers *d*_*1*_, *d*_*2*_ and *d*_*3*_, respectively, are shown in Table 8.

**Table 8:** Three scores matrices of attributes.

Experts’	Alternatives	Attribute		
assessment		*u*_*1*_	*u*_*2*_	*u*_*3*_
*d*_*1*_	*A*_*1*_	(0.80,0)	(0.50,0.30)	(0.50,0.20)
	*A*_*2*_	(0.85,0.01)	(0.85,0.15)	(0.80,0.10)
	*A*_*3*_	(0.99,0.01)	(0.90,0.05)	(0.85,0.05)
*d*_*2*_	*A*_*1*_	(0.10,0.90)	(0.15,0.70)	(0.20,0.60)
	*A*_*2*_	(0.20,0.65)	(0.35,0.60)	(0.30,0.50)
	*A*_*3*_	(0.25,0.01)	(0.50,0.40)	(0.40,0.40)
*d*_*3*_	*A*_*1*_	(0.05,0.95)	(0.20,0.75)	(0.15,0.65)
	*A*_*2*_	(0.15,0.80)	(0.40,0.60)	(0.30,0.60)
	*A*_*3*_	(0.35,0.60)	(0.50,0.40)	(0.35,0.50)

Suppose that the weights *λ*_1_, *λ*_2_ and *λ*_3_ of the DMs *d*_*1*_, *d*_*2*_ and *d*_*3*_ are 0.36, 0.32 and 0.32, respectively. Suppose that the weights of the attributes *u*_*1*_, *u*_*2*_ and *u*_*3*_ given by the DMs are 0.01, 0.49 and 0.50, respectively, i.e., *w*_1_ = 0.01, *w*_2_ = 0.49 and *w*_3_ = 0.50.

**【Step1】:** Based on the decision matrices *d*_*1*_, *d*_*2*_ and *d*_*3*_ and the weights of the attributes given by the DMs, we can get the weighted (on attributes) scores matrices $x_{i}^{k}$ by Eq (7), as shown in Table 9.

**Table 9:** Three weighted (on attributes) scores matrices.

Alternatives	Decision maker		
	*d*_*1*_	*d*_*2*_	*d*_*3*_
*A*_*1*_	(0.505,0)	(0.175,0.650)	(0.174,0.700)
*A*_*2*_	(0.827,0.119)	(0.324,0.548)	(0.350,0.602)
*A*_*3*_	(0.880,0.880)	(0.450,0.386)	(0.4280.449)

**【Step2】:** Based on Eq (8) and Eq (9), we can obtain the range of *γ* as

−4.53≤γ≤6.73

Assume that *γ* =-4, *γ* = 0 and *γ* = 4. The reason why we choose modest values (-4, 0 and 4) is because example 1 has examined the effect of extreme values, and the purpose of example 2 is to examine a modest effect of the variable weight in the permitted range. Then, we can get the weights of the DMs in different situations, as shown in Table 10.

**Table 10:** The weights of DMs in different situations.

Different	Alternatives	DMs’ weights		
parameters		*d*_*1*_	*d*_*2*_	*d*_*3*_
*γ* = −4	*A*_*1*_	0.042	0.447	0.510
	*A*_*2*_	0.392	0.254	0.355
	*A*_*3*_	0.427	0.260	0.313
*γ* = 0	*A*_*1*_	0.360	0.320	0.320
	*A*_*2*_	0.360	0.320	0.320
	*A*_*3*_	0.360	0.320	0.320
*γ* = 4	*A*_*1*_	0.678	0.193	0.130
	*A*_*2*_	0.328	0.386	0.285
	*A*_*3*_	0.293	0.380	0.327

**【Step3】:** Based on Eq (11), we establish the aggregated decision matrix in different situations, as shown in Table 11.

**Table 11:** Three individual weighted (on attributes and DMs) decision matrices.

Different	Alternatives	The aggregated decision matrix
parameters		
*γ* = −4	*A*_*1*_	(0.192,0)
	*A*_*2*_	(0.609,0.273)
	*A*_*3*_	(0.710,0.175)
*γ* = 0	*A*_*1*_	(0.313,0)
	*A*_*2*_	(0.591,0.276)
	*A*_*3*_	(0.678,0.203)
*γ* = 4	*A*_*1*_	(0.416,0)
	*A*_*2*_	(0.573,0.278)
	*A*_*3*_	(0.644,0.235)

**【Step4】:** Based on Eq (12), we rank all the alternatives in different situations, as shown in Table 12.

**Table 12:** A comparison of the orders of the alternatives of Example 2 for different parameters.

Different parameters	*A*_*1*_	*A*_*2*_	*A*_*3*_
*γ* = −4	**3**	**2**	**1**
*γ* = 0	**3**	**2**	**1**
*γ* = 4	**1**	**3**	**2**

Table 13 shows a comparison of the order of the alternatives for different approaches. As shown above, the interesting thing is that with the proposed method, when *γ* = −4 and *γ* = 0, Xu [48]’s method, Zeng and Su [49]’s method and Chen et al. [47]’s method have the same preference order of the alternatives, i.e. *A*_3_>*A*_1_>*A*_2_. In addition, with the proposed method, when *γ* = 4, Yue [41]’s method has the same preference order, i.e. *A*_1_>*A*_3_>*A*_2_. Hence, the calculation result further explains that through the adjustment of the parameters, the proposed method can effectively reflect the various decision results by Xu [48], Yue [41], Zeng and Su [49] and Chen et al. [47]’s methods. As in example 1, the proposed approach also can obtain different decision results by adjusting different parameters. By setting disparate parameters, some of the ranking orders are the same as the existing studies, while some new ranking order are also obtained. Therefore, through the comprehensive analysis of example 1 and example 2, we have once again analyzed the effectiveness of the proposed variable weight based weighting method for DMs in MAGDM problem with IFS.

**Table 13:** A comparison of the orders of the alternatives of Example 2 for different methods.

Methods		Preference order
Xu [48]		*A*_3_>*A*_1_>*A*_2_
Yue [41]		*A*_1_>*A*_3_>*A*_2_
Zeng and Su [49]		*A*_3_>*A*_1_>*A*_2_
Chen et al. [47]		*A*_3_>*A*_1_>*A*_2_
The proposed method	*γ* = −4	*A*_3_>*A*_1_>*A*_2_
	*γ* = 0	*A*_3_>*A*_1_>*A*_2_
	*γ* = 4	*A*_1_>*A*_3_>*A*_2_

## 6 Conclusions

The MAGDM is an important decision tool for complex issues, and intuitionistic fuzzy information is appropriate for dealing with the ambiguities and imprecision inherent in MAGDM problems[41]. Many approaches have been proposed for the solution with different types of decision information. However, in the existing methods, once the weight vector of DMs is computed, its numerical value no longer changes. The weight vector of DMs is always constant. Therefore, this paper presents a novel variable weight vector-based approach to obtain the weights of DMs for MAGDM with IFSs. In this approach, we use the variable weights theory to determine the weights of DMs and add an adjustable parameter to measure the capability of changing weights. Finally, the decision opinions for every alternative offered by each DM is aggregated into a composite assessment value according to the IFWA operator, and the most satisfying alternative is selected.

This proposed method extends the current research in two ways. (1) Unlike most existing studies that treat the weights of DMs as a constant vector [23, 29, 30, 38, 40, 41], this paper treats them as a change vector and proposes a novel method for its determination based on variable weights theory [42, 43]. As above, our approach can effectively reduce the irrationalities on the results of decision-making cause by the constant DMs’ weight vector methods and is very suitable for those decision problem requiring repeated evaluations (e.g., singing contest score). (2) In order to measure the degree of change, we also introduce adjustable parameters to the variable weight vector, and which can comprehensive manifest the preferences of senior managers to DMs, and offers room to adjust for aggregating assessment information when some DMs provide apparentlyunreasonable suggestions. Hence, by taking into account both the changing weights and preferences of the top management to DMs, our approach can make the rank results more rational than the existing subjective and objective weighting approaches for determining DMs’ weights in MAGDM with IFSs.

This study enriches the methodology and theory for determining DMs’ weights in MAGDM with IFSs, and also has below practical implications: (1) Our method takes full advantage of experts. By applying variable weights theory in FMAGDM, our study can effectively avoid irrationalities caused by DM’s knowledge and personal preferences bias. We also add adjustable parameters in decision process, which making the final chosen solution more reasonable and scientific; (2) Our study offers universality and practicability. The analysis of illustrative examples prove the universality and practicability of our approaches, and it can be integrated with other approaches for apply in a variety of decision-making problems, such as partner selection, facility location, etc.

Although the available study results, there is work remaining to be done in the future. First, this study only focuses on the determination of DMs weights in MAGDM, and future work should pay more attention to the determination of attributes weights to make the proposed method more comprehensively. Second, future work should also focuses on develop new aggregation methods to make the evaluation information aggregation phase more efficient. Finally, the language set of this study is in the form of intuitionistic fuzzy numbers, and future work should be implement to support other language sets, such as interval-valued intuitionistic fuzzy numbers, rough sets or vague sets and so on.
